# ε‐poly‐L‐lysine‐modified polydopamine nanoparticles for targeted photothermal therapy of drug‐resistant bacterial keratitis

**DOI:** 10.1002/btm2.10380

**Published:** 2022-08-04

**Authors:** Wenjie Fan, Haijie Han, Zhouyu Lu, Yue Huang, Yin Zhang, Yaoyao Chen, Xiaobo Zhang, Jian Ji, Ke Yao

**Affiliations:** ^1^ Eye Center, the Second Affiliated Hospital, School of Medicine Zhejiang University Hangzhou People's Republic of China; ^2^ Zhejiang Provincial Key Lab of Ophthalmology, the Second Affiliated Hospital, School of Medicine Zhejiang University Hangzhou People's Republic of China; ^3^ MOE Key Laboratory of Macromolecule Synthesis and Functionalization of Ministry of Education, Department of Polymer Science and Engineering Zhejiang University Hangzhou People's Republic of China

**Keywords:** drug‐resistant bacteria, keratitis, photothermal therapy, polydopamine, ε‐poly‐l‐lysine

## Abstract

Bacterial keratitis can lead to intraocular infection and even blindness without prompt and potent treatments. Currently, clinical abuse of antibiotics encouraged the evolution of resistant bacteria. Conventional antibiotic eye drops based keratitis treatment has been heavily restricted due to the lack of bactericidal efficiency and easy induction of bacterial resistance. Hence, developing an effective treatment strategy for bacterial keratitis is of great significance. In this work, we investigated ε‐poly‐l‐lysine (EPL)‐modified polydopamine (PDA) nanoparticles (EPL@PDA NPs)‐mediated antibacterial photothermal therapy (aPTT), to cope with methicillin‐resistant *Staphylococcus aureus* (MRSA)‐induced keratitis. The surface modification of cationic peptide EPL enables EPL@PDA NPs to specifically target negatively charged MRSA and induces local hyperthermia to kill the bacteria under low ambient temperature. Under near‐infrared (NIR) irradiation, the sterilization efficiency of EPL@PDA NPs suspension for MRSA in vitro was up to 99.96%. The EPL@PDA‐mediated aPTT presented potent antibacterial efficacy in treating MRSA‐induced keratitis with little corneal epithelial cytotoxicity and good biocompatibility. In conclusion, the bacterial‐targeting aPTT platform in this work provides a prospective method for the management of MRSA‐induced refractory bacterial keratitis.

## 1 INTRODUCTION

As a purulent corneal infection caused by bacteria, bacterial keratitis can lead to corneal melting, scarring, and perforation if not treated promptly and potently.^1^, ^2^ Conventional clinical protocol includes the topical administration of broad‐spectrum antibiotic eye drops. Whereas the development of novel antibiotics has encountered huge obstacles to keep pace with the evolution of multidrug‐resistant (MDR) bacteria.^3^, ^4^ Selection of sensitive antibiotics requires time‐consuming laboratory tests, which could delay the illness course. Moreover, the toxicity caused by the high dose of antibiotics also needs to be taken into consideration.^5^, ^6^ To address these challenges, novel board‐spectrum therapeutic approaches such as metal nanoparticles, nanozymes, cationic species, photothermal therapy (PTT), and photodynamic therapy (PDT) have been investigated.^7^, ^8^, ^9^, ^10^, ^11^, ^12^, ^13^, ^14^, ^15^

PTT utilizes photothermal transduction agents' (PTAs) photothermal conversion effect to generate heat energy under external light irradiation, and raise the surrounding environment's temperature, to eliminate the target cancer cells, bacteria, and other pathogenic microorganisms.^16^, ^17^, ^18^, ^19^, ^20^ The broad‐spectrum bactericidal efficiency and relatively straightforward sterilization process of PTT have aroused enormous attention. PTT has been widely reported to exhibit excellent performance in managing diseases such as cancer and bacterial infections currently.^21^, ^22^, ^23^, ^24^, ^25^, ^26^ Radiated PTAs can locally raise the temperature (over 50 °C) to cause microbial death by induction of oxidative stress, leading to protein hyperthermia‐denaturation, nucleic acid degradation, and cell membrane destruction.^27^, ^28^ Hence, such physical therapy has a huge natural strength in dealing with bacterial infections caused by MDR strains, such as ampicillin‐resistant *Escherichia coli* (*E. coli*) and methicillin‐resistant *Staphylococcus aureus* (MRSA).^8^, ^29^, ^30^, ^31^

Among a variety of PTAs, polydopamine (PDA) has distinguished itself owing to excellent photothermal conversion efficiency and brilliant biocompatibility compared to traditional metallic nanostructures.^32^ Its photothermal conversion efficiency can reach 40%, which promotes its application in the management of cancer and bacterial infection.^33^, ^34^ Furthermore, PDA's catechol structure is easy to be oxidized to diquinone structure, so it can react with various functional groups (e.g. amine and thiol) via Michael addition and/or Schiff base reaction, and easily be surface modified.^35^, ^36^ Additionally, the preparation of PDA is rather straightforward and requires mild conditions, as dopamine can self‐polymerize to form PDA in a weak alkaline reaction environment.^37^ However, conventional aPTT lacks the specific bactericidal ability, and the hyperthermia produced by aPTT will affect and damage the surrounding tissues during sterilization. To diminish unnecessary tissue lesions and enhance bactericidal efficiency, specific modification of PTAs aiming at improving their targeting ability and concentrating the heat on bacteria has become a feasible strategy.^35^, ^38^, ^39^, ^40^ ε‐poly‐l‐lysine (EPL) is a natural cationic homopolymer containing 25–35 l‐lysine residues. EPL can selectively attach to bacteria surfaces in the physiological environment by electrostatic interaction with the negatively charged cytoplasmic membrane.^41^, ^42^ Moreover, EPL is biodegradable, nontoxic, and more affordable to produce than other cationic peptides.^43^

**SCHEME 1:**
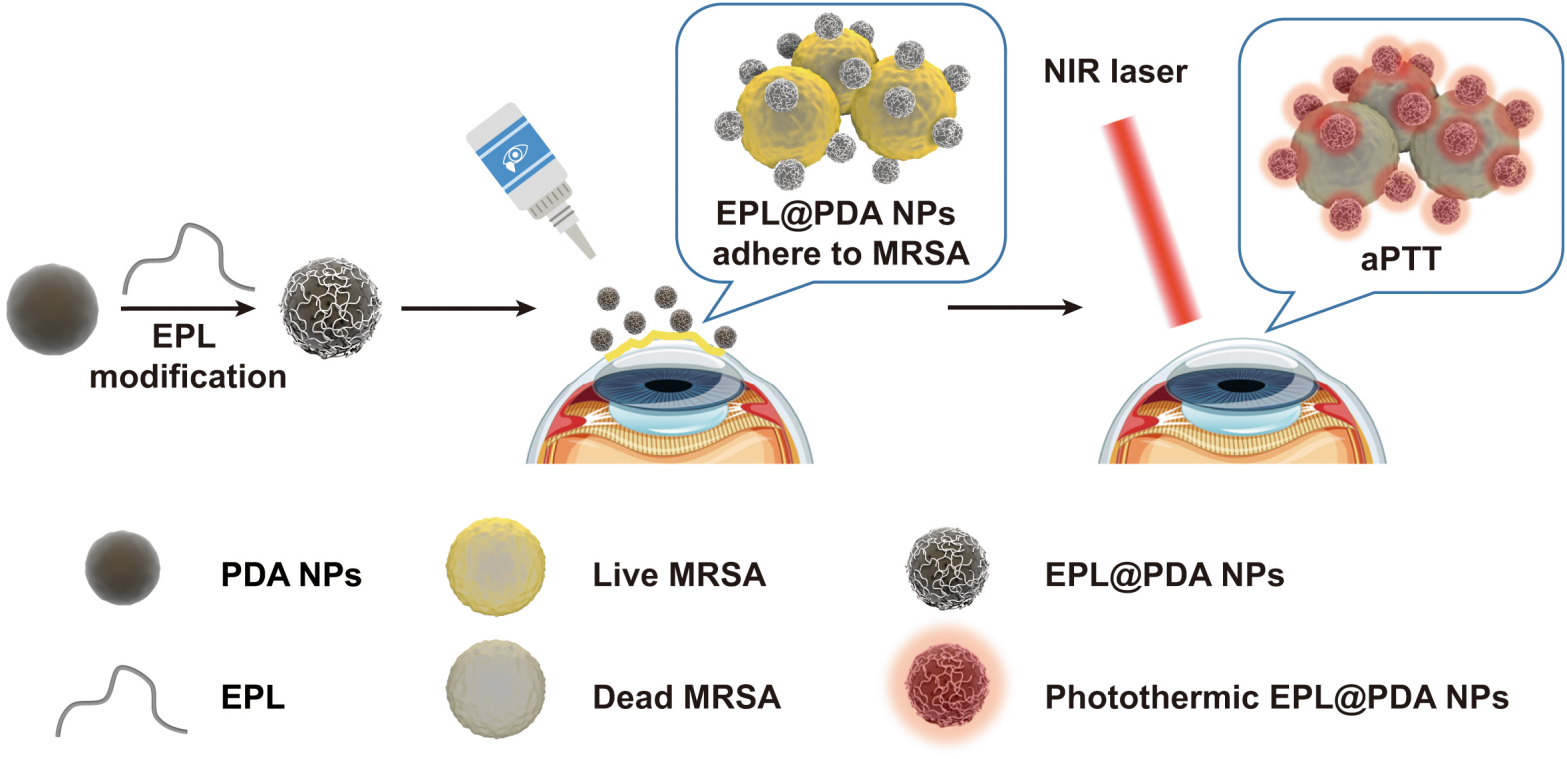
Schematic illustration of EPL@PDA NPs preparation and photothermal sterilization process of MRSA

In this study, EPL‐modified PDA composite nanoparticles (EPL@PDA NPs) based aPTT was developed to achieve low‐temperature sterilization in the management of MDR MRSA‐induced keratitis (Scheme 1). As a cationic peptide, EPL is capable of binding to negatively charged bacterial cytoplasmic membrane by electrostatic interaction in nature.^44^, ^45^ The surface modification of EPL enables EPL@PDA NPs to specifically target negatively charged bacteria. Under the irradiation of NIR lasers (808 nm), EPL@PDA NPs can generate local hyperthermia on the surface of MRSA. The concentration of free EPL@PDA NPs decreased significantly, as they were enriched around bacteria. Therefore, the aPTT platform can efficiently kill the bacteria under low ambient temperature, which avoids thermal damage to surrounding tissues to a large extent. Hence, EPL@PDA NPs mediated aPTT can achieve rapid and efficient eradication of bacteria, providing a novel and prospective method for treating MRSA‐caused corneal infection.

## 2 RESULTS AND DISCUSSION

### 2.1 Preparation and characterization of EPL@PDA NPs

**FIGURE 1:**
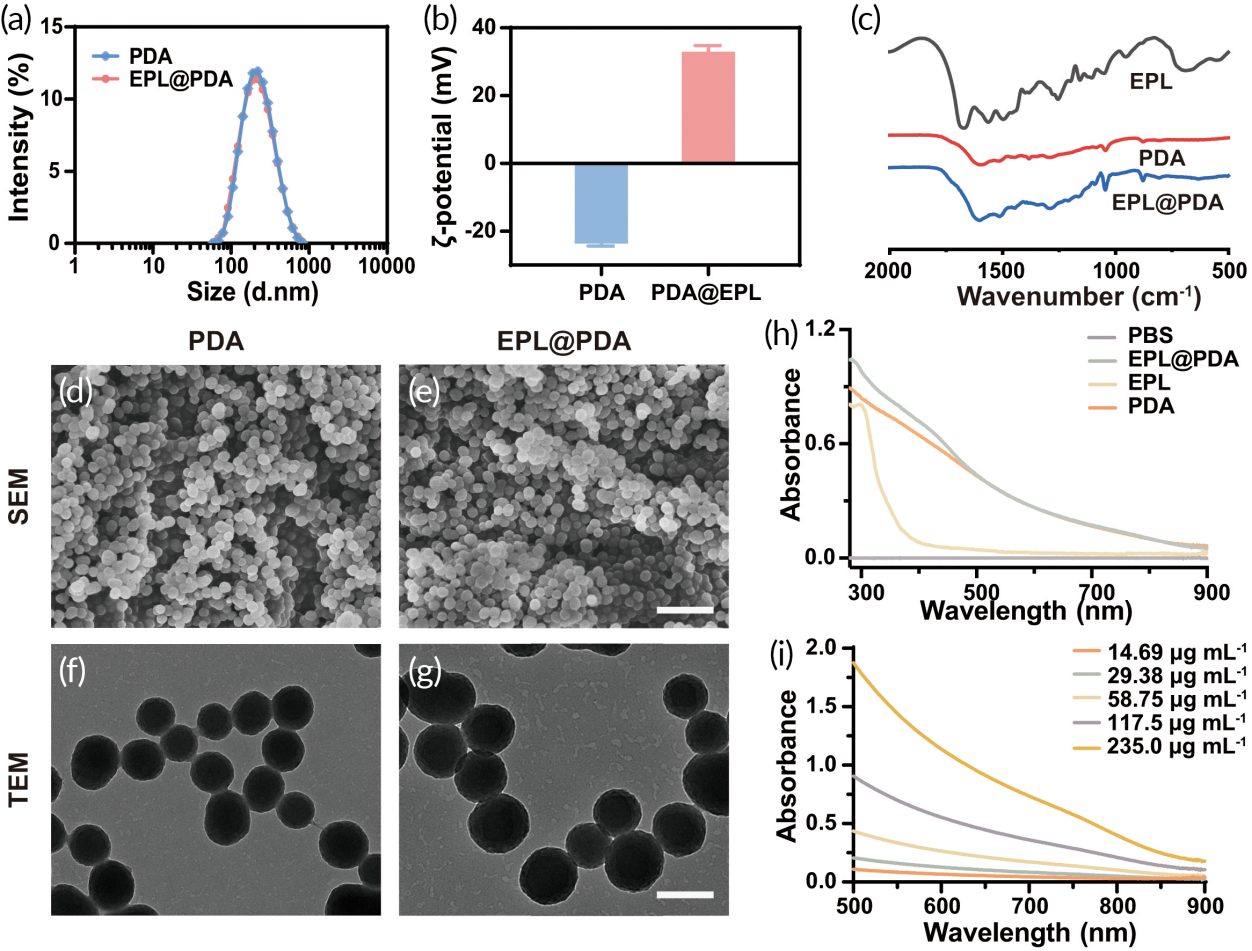
Characterization of PDA and EPL@PDA NPs. Hydrodynamic diameter (a) and ζ‐potential (b) of PDA and EPL@PDA NPs. (c) FT‐IR image of EPL, PDA, and EPL@PDA NPs. SEM images of PDA (d) and EPL@PDA NPs (e). Scale bar: 1 μm. TEM images of PDA (f) and EPL@PDA NPs (g). Scale bar: 200 nm. UV–vis absorbance of EPL, PDA, and EPL@PDA NPs (e) and PDA NPs with different concentrations (f)

PDA NPs were fabricated in weak alkaline aqueous conditions, and subsequent modification of EPL was taken place in an aqueous environment via Michael addition and/or Schiff base reaction. Dynamic light scattering (DLS) was utilized to measure the hydrodynamic diameter of nanoparticles (Figure 1a). EPL@PDA NPs have an average diameter of 203.2 nm with a unimodal size distribution in phosphate‐buffered saline buffer (PBS, pH 7.4). The particle size observed by transmission electron microscopy (TEM) and scanning electron microscope (SEM) was consistent with that measured by DLS, where PDA and EPL@PDA NPs exhibited a regular and uniform spherical morphology (Figure 1d–g). The ζ‐potential of the PDA NPs was −23.2 mV, and the modification of EPL reversed the ζ‐potential to 35.0 mV (Figure 1b). The change of ζ‐potential indicated that EPL chains that possessed abundant cationic amino groups were successfully modified to the surface of PDA NPs. Since PDA and EPL can be covalently conjugated by —C=N—, we further characterized the modification of EPL by Fourier transform infrared spectroscopy (FT‐IR) (Figure 1c). The peak of —C=N— (1598 cm^−1^) indicated that EPL was grafted onto the PDA NPs via Schiff base reaction. Amido bond —CO—NH— (peak at 1255 cm^−1^) existed in EPL, but not in PDA, so the appearance of this peak in the FT‐IR spectroscopy of EPL@PDA NPs ulteriorly indicated the modification. We investigated PDA NPs and EPL@PDA NPs' absorption in the region of 500–900 nm via ultraviolet–visible (UV–Vis) spectrophotometer (Figure 1h,i). Since the absorbance was consistent with concentration change, the linear absorbance‐concentration standard curve of PDA NPs at 808 nm can be drawn, to calibrate the concentration of PDA and EPL@PDA NPs (Figure S1). The coating amount of EPL in EPL@PDA can be calculated as 7.9 μg per 100 μg EPL@PDA accordingly.

### 2.2 Photothermal conversion performance of EPL@PDA NPs

**FIGURE 2:**
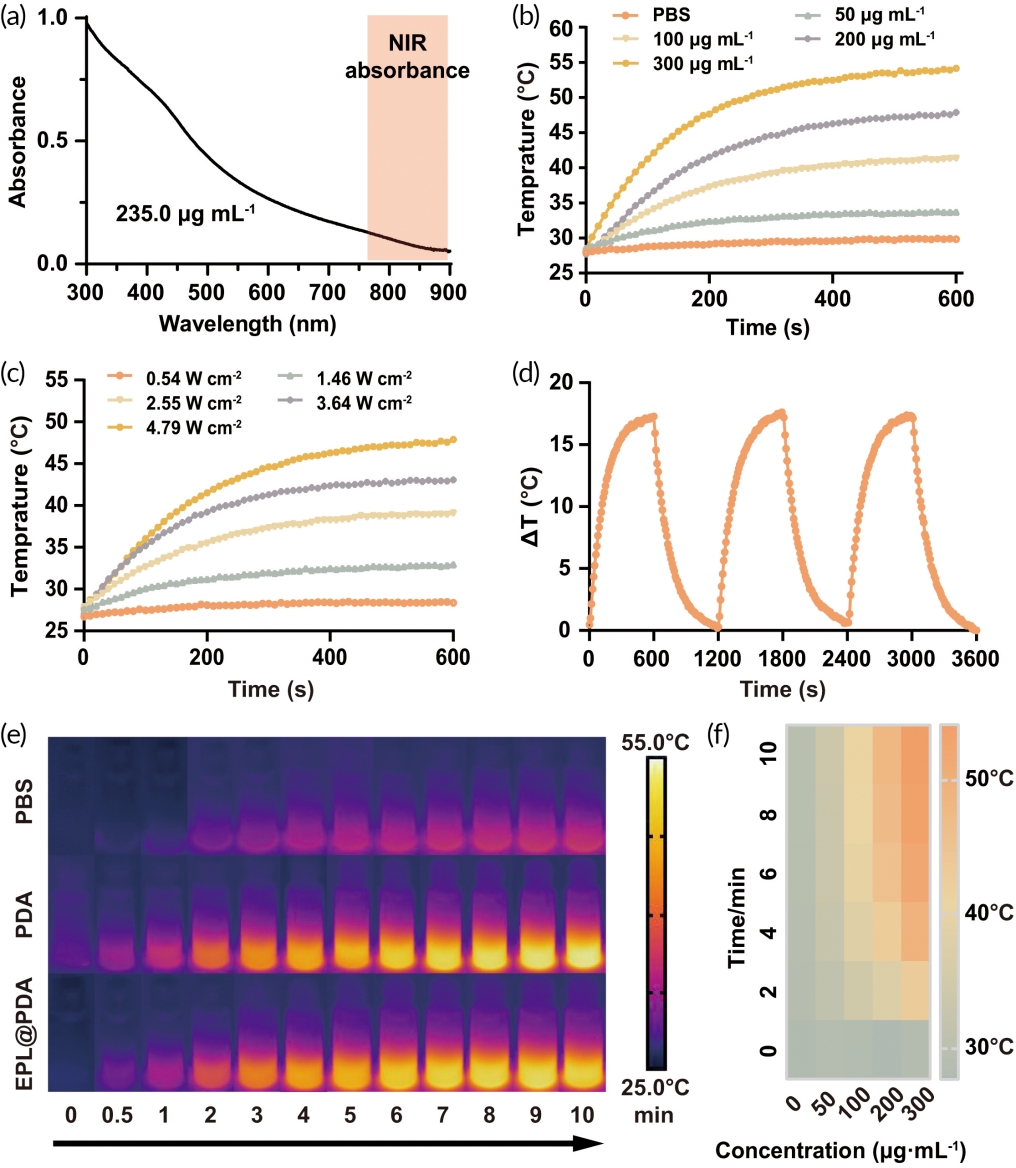
Photothermal conversion evaluation of EPL@PDA NPs. (a) UV–vis spectra of EPL@PDA NPs (235.0 μg mL^−1^). Photothermal temperature changing curves of EPL@PDA NPs at concentrations from 0 to 200.0 μg mL^−1^ under the NIR intensity of 3.64 W cm^−2^ (b), at 200.0 μg mL^−1^ under intensity from 0.54 to 4.79 W cm^−2^ (c), and at 200.0 μg mL^−1^ in a cyclic photothermal experiment where NIR light (3.64 W cm^−2^, 10 min) are turned on and off for 3 times (d). (e) Corresponding thermographic images of PBS, PDA, and EPL@PDA NPs suspensions (200.0 μg mL^−1^) under irradiation (4.79 W cm^−2^, 10 min). (f) Thermal vision parade of different EPL@PDA NPs concentrations under 10‐min irradiation

PDA NPs display absorption in the NIR region (760–900 nm) (Figure 2a). In this work, NIR laser (808 nm) was exploited to investigate EPL@PDA NPs' photothermal conversion capability. We explored the relationship between laser intensity, concentration, irradiation time, and temperature rising degree. Specifically, EPL@PDA NPs in PBS were irradiated by 808 nm NIR light for 10 min, respectively. The photothermal activity of EPL@PDA NPs (concentration from 50 to 300 μg mL^−1^) was investigated by comparing the temperature change when exposed to NIR light (3.64 W cm^−2^). EPL@PDA NPs suspensions displayed a significant temperature rise, compared to the negative control (PBS), and exhibited concentration‐dependent properties (Figure 2b). Analogously, laser intensity‐dependent photothermal effect was also observed when EPL@PDA NPs solution (200 μg mL^−1^) was irradiated with different powers (0.54–4.79 W cm^−2^) (Figure 2c). Such concentration and laser intensity‐dependent activities provided the feasibility of selecting the conditional parameters for subsequent in vitro and in vivo antibacterial evaluations (Figure 2e). Under the irradiation of NIR light (4.79 W cm^−2^, 10 min), EPL@PDA NPs suspension (200 μg mL^−1^) reached approximately 54.1°C, indicating that EPL@PDA NPs maintained brilliant photothermal properties after the modification. In addition, a cyclic photothermal experiment was conducted to investigate the photostability of EPL@PDA NPs (Figure 2d). In the rising‐cooling circles, the range of temperature exhibited negligible change, indicating excellent photothermal stability of EPL@PDA NPs. Thermographic images of PBS, PDA, and EPL@PDA NPs suspension (200.0 μg mL^−1^) under NIR irradiation (808 nm, 4.79 W cm^−2^, 10 min) and corresponding thermal vision were displayed in Figure 2e,f. Hence, the high photostability and excellent photothermal conversion performance of EPL@PDA NPs inspired further antimicrobial use.

### 2.3 Selective adhesion of EPL@PDA NPs on bacterial membrane

**FIGURE 3:**
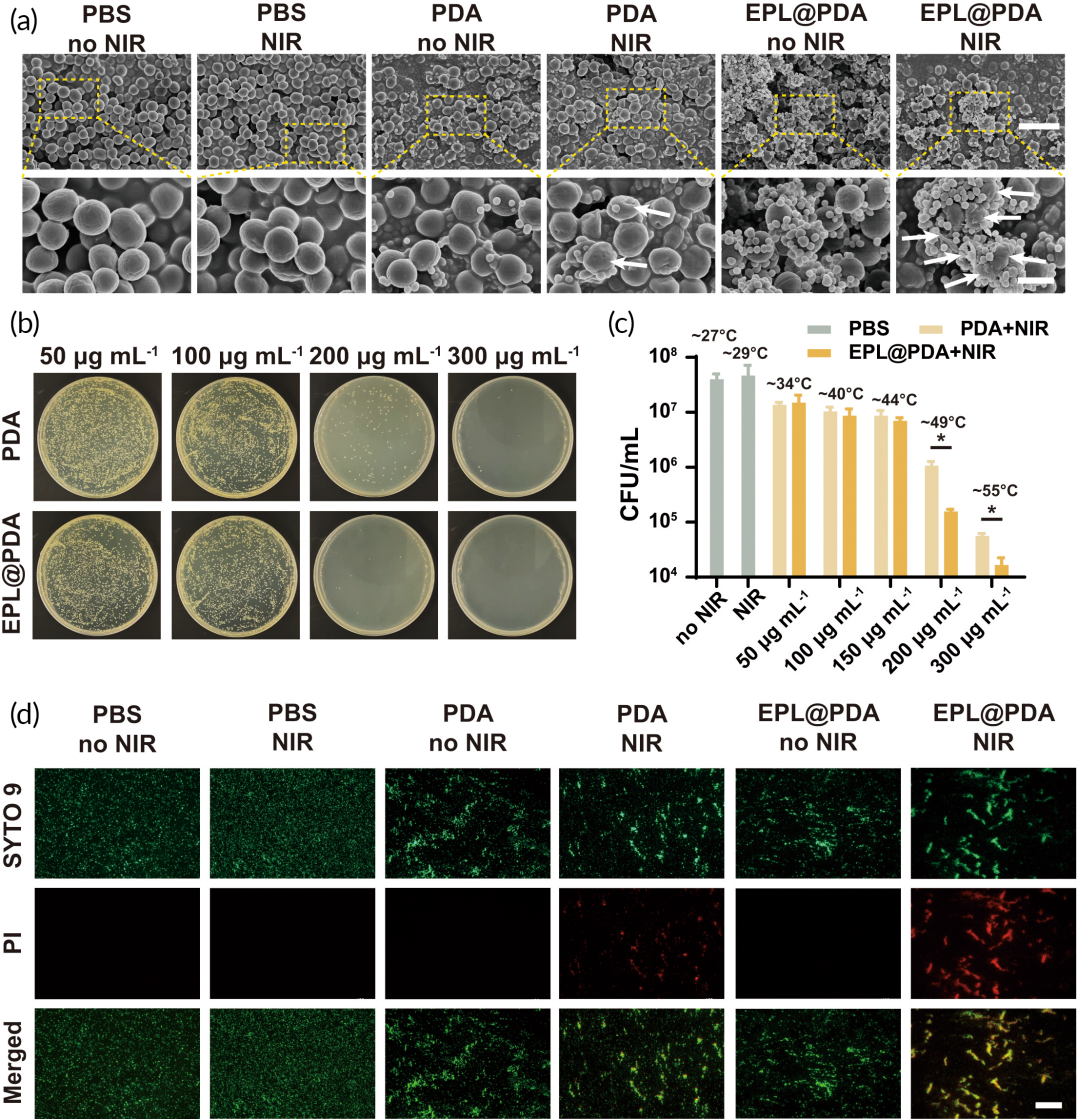
In vitro bactericidal performance of EPL@PDA NPs. (a) SEM photographs of MRSA incubated with PBS, PDA NPs, or EPL@PDA NPs and irradiated with 808 nm laser (3.64 W cm^−2^, 10 min) or not. Scale bar: 3 μm. Scale bar of enlarged images: 1 μm. (b) Photographs of standard plate counting assay after different treatments, where MRSA was administrated with PDA or EPL@PDA NPs and exposed to 808 nm light (3.64 W cm^−2^) for 10 min. (c) Bacterial survival rate of MRSA treated with PBS (under NIR irradiation or not), or PDA and EPL@PDA NPs in different concentrations under NIR irradiation. (d) Fluorescence microscopic visualization of MRSA after the adoption of Live/Dead staining. The green fluorescence (SYTO 9) represents live bacteria and the red fluorescence (PI) indicates dead bacteria. Scale bar: 150 μm. Results were presented as the mean ± SD. *p < 0.05. Significance was calculated by *t*‐test

It is well known that PDA NPs‐based aPTT displayed satisfactory bactericidal effect. However, the general temperature rise may cause unwelcome damage to affected tissues. Developing bacteria‐targeting PTAs to achieve sterilization under a low temperature could improve therapeutic efficiency and reduce side effects.^18^ As EPL@PDA NPs were fabricated to be positively charged, they were inclined to adhere to the bacteria. SEM was utilized to observe and characterize the selective adhesion between EPL@PDA NPs and MRSA. After PBS, PDA and EPL@PDA NPs (200 μg mL^−1^, 100 μL) were incubated with 100 μL 5 × 10^7^ colony forming units ml^−1^ (CFU mL^−1^) MRSA suspension for 1 h, respectively, the mixed suspensions were either exposed to 808 nm NIR laser (3.64 W cm^−2^, 10 min) or not. The quantity of EPL@PDA NPs adhered to MRSA significantly exceeded PDA NPs, suggesting a strong bacterial targeting activity of EPL@PDA NPs (Figure 3a). After the mixed suspensions were irradiated, MRSA incubated with EPL@PDA NPs exhibited evident condensation and deformation. The demonstration indicated that the produced hyperthermia could cause a strong disturbance to the bacterial membrane and subsequently result in cell death.

### 2.4 In vitro bactericidal performance to MDR bacteria

The antibacterial performance of EPL@PDA NPs mediated aPTT against MRSA was investigated via the typical spread plate method and dilution‐plate method (Figure 3b,c). Mixed suspension of PBS, PDA and EPL@PDA NPs (50–300 μg mL^−1^) and MRSA (5 × 10^7^ CFU mL^−1^) were incubated for 1 h, respectively. NIR irradiation groups were exposed to 808 nm NIR laser (3.64 W cm^−2^, 10 min). When PBS was incubated with MRSA with or without irradiation, the CFU of bacteria showed negligible change, indicating that the laser itself possessed few impacts on sterilization (Figure 3c). The bactericidal activity of PDA and EPL@PDA with NIR irradiation was compared in Figure 3b,c. When the NIR laser was employed, PDA NPs effectively converted the light energy to heat energy and raised the temperature of the solution, as the sterilization rate of PDA NPs was up to 97.33% (200 μg mL^−1^) and 99.86% (300 μg mL^−1^), respectively. Additionally, modification of EPL enabled PDA NPs to generate hyperthermia at the surface of MRSA. Under NIR irradiation, the temperature of MRSA surface to which EPL@PDA NPs attached can be estimated to reach 100°C before the heat balance.^33^ Such local high temperature can induce a significant bactericidal effect, and the sterilization rate was up to 99.61% (200 μg mL^−1^) and 99.96% (300 μg mL^−1^) while the general temperature of the suspension remained at a relatively low level (~49°C and ~55°C). The 300 μg mL^−1^ EPL@PDA NPs suspension displayed almost no bactericidal effects (Figure S2). Besides, there was no significant difference in bacteria viability even under 47.3 μg mL^−1^ EPL (corresponding EPL@PDA NPs 600 μg mL^−1^), illustrating that cationic peptide EPL has a limited killing effect against MRSA. The antibacterial performance of EPL@PDA NPs (without NIR irradiation) and EPL further eliminated the interference of EPL's intrinsic bactericidal activity. The results of Live/Dead BacLight assay (Figure 3d) were also consistent with the result of the plate counting method mentioned above. The green fluorescence (SYTO 9) represented live bacteria and the red fluorescence (PI) represented dead bacteria. The interaction between the nanoparticles and the MRSA could induce bacteria congregation to some extent, but this did not affect the results of the experiment. When MRSA was mixed with EPL@PDA NPs and exposed to NIR, they displayed the strongest PI fluorescence. Hence, we believe that the modification of EPL significantly enhanced the sterilization efficiency of aPTT.

### 2.5 In vivo anti‐infective performance on MRSA‐infected bacterial keratitis model

**FIGURE 4:**
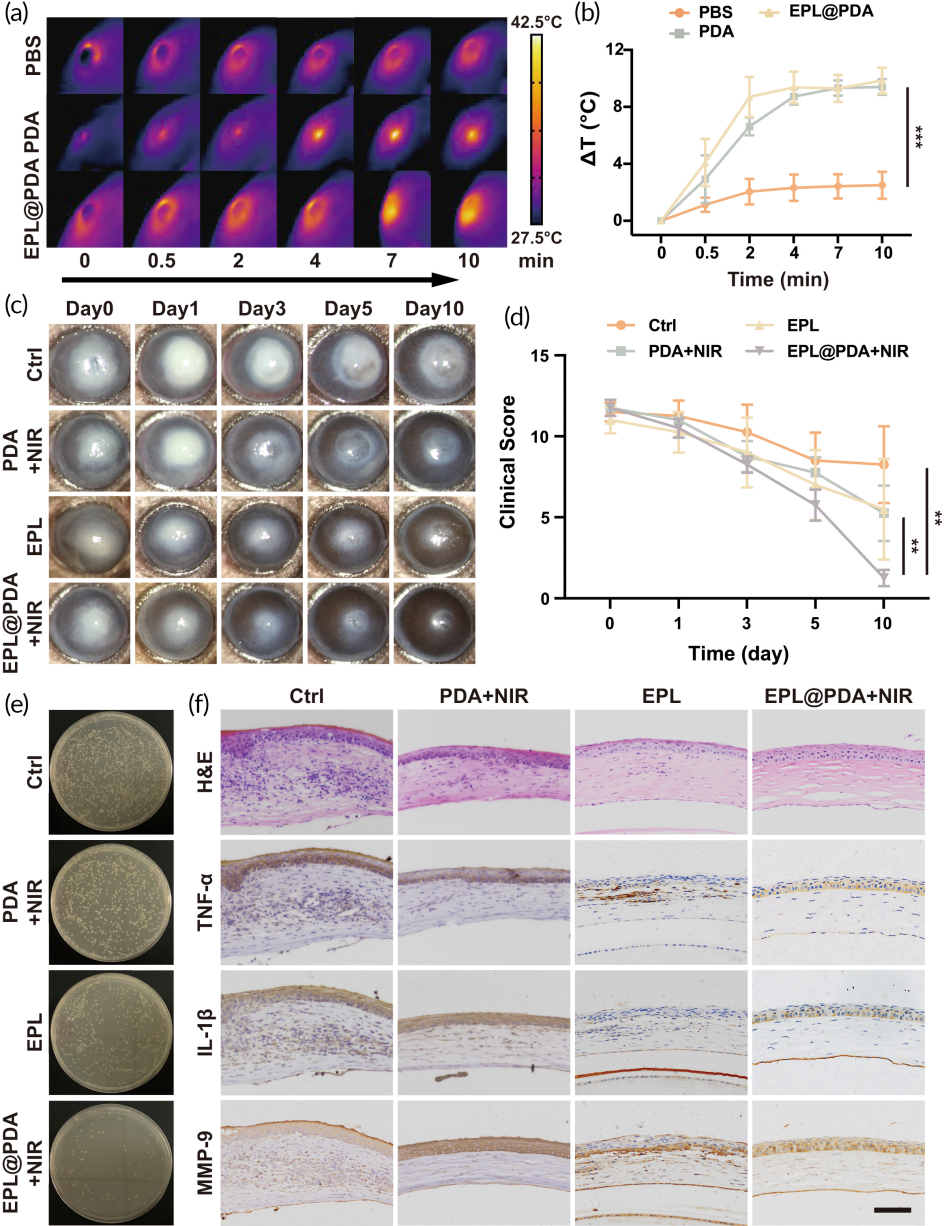
In vivo bactericidal performance of EPL@PDA NPs. Infrared thermography of murine cornea when treated with PBS, PDA NPs, and EPL@PDA NPs under 808 nm laser irradiation (2.55 W cm^−2^, 10 min) (a) and corresponding temperature change curve (b). (c) Representative slit‐lamp photomicrographs of MRSA‐induced keratitis treated with PBS, PDA + NIR irradiation, EPL, and EPL@PDA + NIR irradiation. (d) Corresponding clinical scores were evaluated (0–12) according to opacity area, surface regularity, and opacity density. (e) Photographs of MRSA colonies from the mice corneal tissues. (f) Histopathologic and immunohistochemical evaluation of mice corneas on Day 5 after staining with H&E, TNF‐α, IL‐1β, and MMP9. Scale bar: 50 μm. Results were presented as the mean ± SD. *P < 0.05, **P < 0.01, ***P < 0.001. Significance was calculated by one‐way ANOVA

Previous results have proved the brilliant antibacterial ability of EPL@PDA NPs based aPTT platform, which inspired us to further explore its clinical transformation potential for the management of intractable bacterial keratitis. Therefore, we investigated its therapeutic efficacy on a murine MRSA‐induced keratitis model. After the keratitis model was established, an oval focal abscess with a gray‐white‐ish corneal stroma infiltration with obvious boundaries could be observed and recorded via slit lamp (Figure 4c). Each group was given the corresponding treatment. During the aPTT process, the local temperature rose to 41.5°C (PDA) and 41.8°C (EPL@PDA) within the cornea (Figure 4a,b). Owing to the bacterial target effect of EPL@PDA NPs, we were able to conduct the aPTT progress even under such a low temperature without compromising therapeutic efficiency.

The evaluation of therapeutic effect was mainly based on the area and depth of abscess, specifically manifested as the scope and transparency of cornea opaque.^46^ Due to the heavy bacteria burden, the stromal lesions in the Ctrl group shrank slowly, and corneal transparency improved slightly on Day 10. The Ctrl group did not represent a full recovery, which may cause the problem of corneal scarring and consistent visual impairment. Compared to other groups, the EPL@PDA group exhibited the fastest lesion reduction. On Days 1 and 3, the limbal region of the cornea showed promotion of transparency (Figure 4c). Cheerfully, the therapeutic effect of EPL@PDA mediated aPTT could be observed after 10‐day‐treatment, demonstrating a statistical difference among the groups (Figure 4d). In addition, aPTT significantly reduced bacteria burden, according to the corneal bacterial plate culture result (Figure 4e).

Corneal inflammation caused by bacterial infection can be a great catastrophe for the structure of stromal collagen. Since corneal inflammation is the main cause of corneal nebula and other sequelae, we further investigate the severity of corneal inflammation by characterizing the inflammatory factors such as tumor necrosis factor‐α (TNF‐α), interleukin‐1β (IL‐1β), and matrix metalloproteinase‐9 (MMP‐9) in murine cornea. According to the hematoxylin–eosin (H&E) staining and immunohistochemical staining results (Figure 4f), the slice of Ctrl group showed irregularly arranged corneal epithelial cells, stromal edema, and massive infiltration of inflammatory cells such as neutrophils. The expression of inflammatory cytokines such as TNF‐α, IL‐1β, and MMP‐9 was rather high in the untreated cornea, indicating a severe inflammatory response. In contrast, all the treatments exhibited effectiveness in relieving inflammation and restoring the regularity of corneal structures. Among all the treatments, EPL@PDA‐mediated displayed its superiority, as the inflammatory responses were mild, and the three‐layer structure of the cornea was basically returned to normal in terms of histology. The EPL@PDA‐mediated specific aPTT has a potent bactericidal effect, which leads to faster lesion shrinkage and brilliant restoration of cornea function. Besides, the aPTT process did not induce histologic damage owing to the low therapeutic temperature. The results demonstrate a commendable therapeutic effect of EPL@PDA NPs based low‐temperature aPTT, which further reveals its potential for clinical transformation.

### 2.6 Biotoxicity evaluation

The biocompatibility of materials directly determines whether clinical translation can be carried out.^47^ Human corneal epithelial cells (HCECs) were chosen for in vitro cytotoxicity evaluation. The high cell viability of HCECs indicated the excellent biocompatibility of EPL@PDA NPs (Figure S3,4). Besides, fluorescein sodium staining results demonstrated that neither the EPL@PDA NPs nor the temperature increase would damage the corneal epithelium (Figure S5). It can be concluded that EPL@PDA NPs‐based low‐temperature aPTT has a high safety profile for the eye.

**FIGURE 5:**
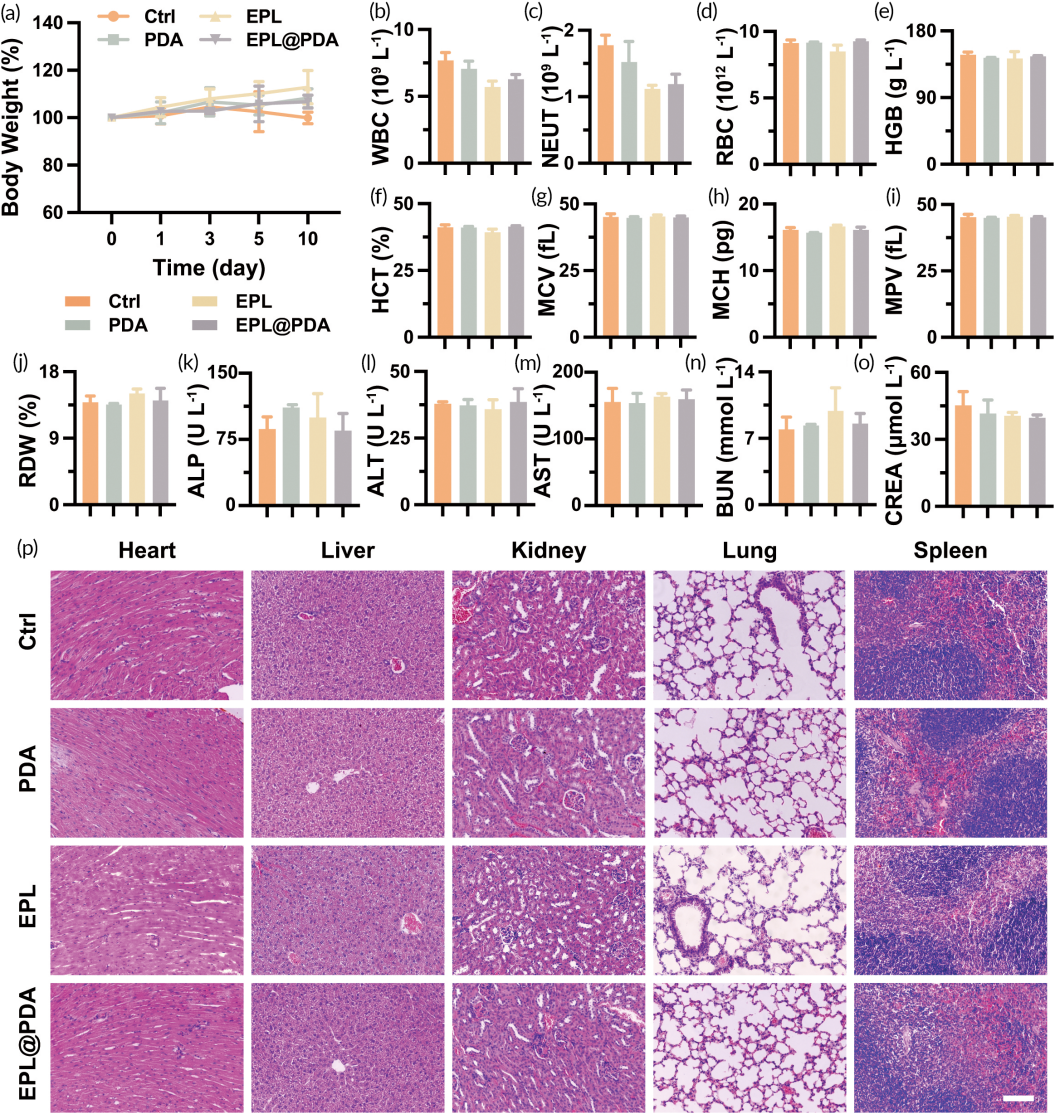
Biotoxicity evaluation. (a) Murine body weight changes throughout the treatment period. Blood routine examination, including WBC (b), NEUT (c), RBC (d), HGB (e), HCT (f), MCV (g), MCH (h), MPV (i), and RDW (j). Blood biochemistry of the liver and kidneys, including ALP (k), ALT (l), AST (m), BUN (n), and CREA (o). (p) Histological evaluation via H&E staining of mice heart, liver, kidney, lung, and spleen after various treatments on Day 10. Scale bar: 100 μm

The mice maintained their weight during the 10‐day‐treatment (Figure 5a). Besides, there were no noticeable abnormalities in the hematology examination, including white blood cell (WBC), neutrophil (NEUT), red blood cell (RBC), hemoglobin (HGB), hematocrit (HCT), mean corpuscular volume (MCV), mean corpuscular hemoglobin (MCH), mean platelet volume (MPV), and red blood cell distribution width (RDW) (Figure 5b–j). Also, alkaline phosphatase (ALP), alanine transferase (ALT), aspartate transferase (AST), blood urea nitrogen (BUN), and creatinine (CREA) remained within the normal range, indicating well‐preserved liver and kidney functions after aPTT and other treatments (Figure 5k–o). H&E staining was conducted on tissue sections of the main murine organs, namely the heart, liver, kidney, lung, and spleen (Figure 5p). Negligible damage was observed in the tissue morphology of the mouse organs. The excellent biocompatibility of the aPTT platform is mainly owing to the low toxicity of EPL@PDA. Besides, low dosing frequency diminishes the amount of the drug circulating throughout the body, which further reduces drug‐induced tissue damage. Therefore, the EPL@PDA‐based aPTT platform displays low biotoxicity, indicating its potential for clinical transformation in managing bacterial keratitis.

## 3 CONCLUSION

In summary, EPL@PDA NPs were fabricated to conduct a targeted aPTT‐mediated antibacterial process and effectively manage MRSA‐induced keratitis under low ambient temperature. In this platform, the negative charge of PDA NPs was reversed by EPL modification. Hence, the positively charged EPL@PDA NPs were able to pose electrostatic interaction toward the plasma membrane of MRSA and selectively enriched nearby the bacteria. Under NIR irradiation, EPL@PDA NPs can raise the temperature to kill the bacteria through a photothermal conversion process. The excellent adhesion activity enables the production of localized hyperthermia, which promotes sterilization efficiency and limits undesirable damage to adjacent tissues. In addition, the excellent biocompatibility of EPL@PDA NPs and less dosing frequency further improved the application in vivo. As a prospective low‐temperature aPTT platform, it displays a huge potential to manage intractable bacterial keratitis.

## 4 EXPERIMENTAL SECTION

### 4.1 Materials

Dopamine hydrochloride was supplied by Sigma‐Aldrich (Shanghai, China). EPL (M_w_ 3000–5000) was supplied by Zhengzhou Bainafo Bioengineering Co., Ltd (Zhengzhou, China). Ammonia solution and ethanol absolute were provided by Sinopharm Chemical Reagent Co., Ltd (Shanghai, China). The 20× PBS buffer was purchased from Sangon Biotech Co., Ltd (Shanghai, China). Deionized water (DI, Millipore Milli‐Q grade) was used in all experiments. Other solvents and reagents were analytical grade and directly used without further purification.

### 4.2 Preparation of PDA and EPL@PDA NPs

The 45 mL of deionized water, 25 mL of ethanol, and 1.5 mL of ammonia solution were added into a 100 mL round‐bottom flask and stirred at 750 rpm for 30 min, providing alkaline conditions and retarder. Then dissolve 250 mg dopamine in 5 mL of deionized water to prepare a dopamine aqueous solution and add it to the flask. After 24 h of continuous stirring (750 rpm), weak alkaline PDA suspension was obtained. The PDA suspension was repeatedly centrifuged and dispersed by ultrafiltration (MWCO = 3000) until the suspension became neutral, in order to terminate the self‐polymerization and condense the suspension.

The PDA NPs suspension (940 mg mL^−1^, 20 mL) and EPL solution (40 mg mL^−1^, 1 mL) were mixed in 50 mL round‐bottom flask. After 24 h stirring (750 rpm), EPL@PDA NPs suspension was acquired and then centrifuged by repeatedly ultrafiltration (MWCO = 50,000) and dispersed by deionized water to remove unreacted EPL.

### 4.3 Characterization of PDA and EPL@PDA NPs

Prepare PDA and EPL@PDA NPs PBS buffer suspension (pH = 7.4), and measure the hydrodynamic diameter and the ζ‐potential of the NPs by Zetasizer Nano‐ZS (Malvern Instruments, UK). A Fourier transform infrared spectroscopy (FTIR) spectrometer (Nicolet 6700, Themo Fisher Scientific LLC, USA) was used to detect the FT‐IR spectrum. Cold field emission SEM (SU8010; Hitachi, Japan) and TEM (HT7700; Hitachi, Japan) were utilized to observe the morphology of PDA and EPL@PDA NPs. The absorbance spectrum of NPs suspension was measured via ultraviolet–visible (UV–Vis) spectrophotometer (UV‐2550; Shimadzu, Japan) to calibrate the concentration of the suspension.

### 4.4 Photothermal conversion ability of EPL@PDA NPs

As for the evaluation of EPL@PDA NPs' photothermal conversion performance, an 808 nm wavelength laser (LSR808H47W, Lasever Inc., China) was exploited to provide a NIR laser, and an infrared thermal imaging camera (FLIR E60, Flir System. Inc., USA) was employed to measure real‐time temperature. Prepare a series of gradient EPL@PDA NPs suspensions (50, 100, 200, 300 μg mL^−1^) in sample bottles and set them aside. The concentration of EPL@PDA NPs was calibrated according to the concentration of PDA in the suspension. Precisely, irradiate each sample bottle with a NIR laser at various light intensities (0.54, 1.46, 2.55, 3.64, 4.79 W cm^−2^) for 10 min and record the thermographic images and temperature change at the same time. Besides, the photothermal stability was evaluated by recording threee photothermic temperature rising‐cooling circles of EPL@PDA NPs suspension. First, expose EPL@PDA NPs suspension (200 μg mL^−1^) to NIR light (3.64 W cm^−2^) for 10 min, then turned off the light for 10 min. After that, turn on the light, and repeat the on–off process for another two times. The temperature was detected and recorded during this heating and cooling process.

### 4.5 In vitro interaction of EPL@PDA NPs with MRSA

MRSA (ATCC 44300) was incubated at 37°C in tryptone soy broth medium (TSB). The bacteria suspension was centrifuged and resuspended three times with a sterile PBS buffer solution for storage. Before use, the concentration of MRSA suspension was detected by OD_595_ (Thermo Fisher Scientific, Waltham, USA), and the suspension was later diluted to 5 × 10^7^ CFU mL^−1^.

MRSA suspension (5 × 10^7^ CFU mL^−1^, 100 μL) was mixed and incubated with PDA or EPL@PDA NPs PBS suspensions (200 μg mL^−1^, 100 μL) for 1 h (37°C, 150 rpm) and irradiated with NIR laser (808 nm, 3.64 W cm^−2^, 10 min). Subsequently, the mixed suspensions were added onto the silicon wafer and deposited for 8 h and later fixed by 2.5% glutaraldehyde for 4 h. Then, alcohol series (10%, 20%, 40%, 60%, 80%, 100%, and 100%) were employed to dehydrate the samples. Finally, the samples were observed by SEM.

### 4.6 In vitro photothermal bactericidal activity

PDA, EPL@PDA NPs PBS suspension (100 μL) were mixed with MRSA suspension (5 × 10^7^ CFU mL^−1^, 100 μl) in a 96‐well plate to obtain mixed suspension of PDA and EPL@PDA NPs with gradient concentration (50, 100, 150, 200, 300 μg mL^−1^). Then, the suspensions were incubated for 1 h (37°C, 150 rpm), were either irradiated by NIR light (808 nm, 3.64 W cm^−2^) for 10 min or left unirradiated. PBS was introduced as a control. Subsequently, the suspension was appropriately diluted with sterile PBS and cultured on Luria‐Bertani (LB) solid medium at 37°C for 12 h. Then assess the bactericidal ability by further standard plate counting assays.

### 4.7 In vivo evaluation of bactericidal efficacy on MRSA‐infected keratitis model

All animal experiments were complied with the Association for Research in Vision and Ophthalmology Statement for the Use of Animals in Ophthalmic and Vision Research and the guidelines for Animal Care and Use Committee, Zhejiang University. All animal experiments were approved by the Animal Ethics Committee, the Second Affiliated Hospital, School of Medicine, Zhejiang University (Approval number: AIRB‐2021‐814). We constructed an MRSA‐infected keratitis model on 6‐week‐old healthy female C57BL/6 mice (~17 g, supplied by the animal center of Zhejiang Academy of Medical Sciences.) to investigate the therapeutic effect of EPL@PDA NPs‐mediated aPTT in vivo. In brief, MRSA (1 μL, 1 × 10^7^ CFU mL^−1^) was injected into the murine corneal stroma. After 16 h of inoculation, the murine cornea was observed using a slit lamp, and the emergence of opacity and edema prompts a successful establishment of the bacterial keratitis model (Day 0). Infected mice were randomly divided into four groups and treated with different regimens, namely Ctrl (PBS), PDA (PDA NPs, 300 μg mL^−1^, NIR), EPL (EPL, 1%), and EPL@PDA (EPL@PDA NPs PBS solution, 300 μg mL^−1^, NIR). In the PTT process, PDA or EPL@PDA NPs suspension was dropped on the ocular surface three times (once every 2 min). Then, irradiate the eye for 5 min using 808 nm (2.55 W cm^−2^) laser once. Subsequently, the progression of the keratitis is observed using slit lamp and recorded by camera, and the mice were sacrificed on Day 10. Then, harvest the eyeballs for corneal homogenate plate counting, histological morphology observation, and extract the main organs (namely heart, liver, kidney, lung, and spleen) to observe the toxicity that treatment regimens exert on mice.

## AUTHOR CONTRIBUTIONS

**Wenjie Fan:** Conceptualization (lead); data curation (lead); investigation (lead); methodology (lead); writing – original draft (lead). **Zhouyu Lu:** Data curation (equal); investigation (equal); writing – review and editing (equal). **Yue Huang:** Data curation (equal); investigation (equal). **Yin Zhang:** Investigation (equal). **Yaoyao Chen:** Investigation (equal). **Xiaobo Zhang:** Investigation (supporting). **Ke Yao:** Conceptualization (equal); funding acquisition (lead); supervision (equal); writing – review and editing (equal). **Jian Ji:** Funding acquisition (lead); supervision (lead); writing – review and editing (equal). **Haijie Han:** Conceptualization (equal); data curation (equal); funding acquisition (lead); investigation (lead); methodology (equal); writing – review and editing (lead).

## Supporting information


**Appendix S1** Supporting Information

## Data Availability

Research data are not shared.
