# Supplementary material for: ε‐poly‐L‐lysine‐modified polydopamine nanoparticles for targeted photothermal therapy of drug‐resistant bacterial keratitis
Source: Bioeng Transl Med. 2022 Aug 4;8(1):e10380. doi: 10.1002/btm2.10380 (PMC9842021; doi:10.1002/btm2.10380)
Supplement: Supplementary file 1 — Appendix S1 Supporting Information [file BTM2-8-e10380-s001.docx]

### ε-poly-L-lysine-modified polydopamine nanoparticles for targeted photothermal therapy of drug-resistant bacterial keratitis

**Wenjie Fan,^a,b,c,#^ Haijie Han,^a,b,#,^*** **Zhouyu Lu,^a,b^** **Yue Huang,^c^ Yin Zhang,^a,b^ Yaoyao Chen,^a,b^ Xiaobo Zhang,^a,b^ Jian Ji,^c,^* Ke Yao^a,b,^***

a Eye Center, the Second Affiliated Hospital, School of Medicine, Zhejiang University, 88 Jiefang Road, Hangzhou 310009, P. R. China;

b Zhejiang Provincial Key Lab of Ophthalmology, the Second Affiliated Hospital, School of Medicine, Zhejiang University, 88 Jiefang Road, Hangzhou 310009, P. R. China;

c MOE Key Laboratory of Macromolecule Synthesis and Functionalization of Ministry of Education, Department of Polymer Science and Engineering, Zhejiang University, Hangzhou 310027, P. R. China;

# These authors contributed equally to this work.

*Corresponding author. E-mail address: xlren@zju.edu.cn (Ke Yao); jijian@zju.edu.cn (Jian Ji); hanhj90@zju.edu.cn (Haijie Han).

1. Materials.

Human cornea epithelium cells (HCECs) were supplied by American Type Culture Collection. DMEM/F12 was obtained from Corning Inc. Calcein-AM and propidium iodide (PI) were both purchased from Beyotime Biotechnology Co., Ltd (Shanghai, China). Other solvents and reagents were analytical grade and directly used without further purification.

1. Experimental Methods.
   1. Live/Dead Assay

DMEM/F12 with 10% fetal bovine serum (FBS), 1% penicillin, and streptomycin was used for the culture of HCECs. HCECs were cultured in 12-well plates at a density of 1.5×10^5^ cells per well with 900 μL of DMEM/F12 overnight. After PDA or EPL@PDA NPs were added to each well (equivalent final NPs concentration of 400 μg mL^-1^) and incubated for 24 h, HCECs were washed three times with PBS. After that, cells were stained with Calcein-AM (at a final concentration of 0.67 μmol L^-1^) and PI (at a final concentration of 50 μg mL^-1^) at 37°C for 30 min, after which the cells were washed with PBS 3 times (2 mL per well). The images of the cells were acquired immediately and analyzed by using a fluorescence microscope (Olympus IX51, Japan).

- 1. Cytotoxicity Assay

The cytotoxicity of PDA and EPL@PDA NPs was determined by the cell counting kit-8 (CCK-8) method. Briefly, HCECs in 90 μL medium were inoculated to 96-well plates at a density of 5 ×10^3^ cells per well overnight, and then cultured for 24 h with PDA or EPL@PDA NPs (equivalent final NPs concentration of 0, 25, 50, 100, 200, 300, and 400 μg mL^-1^) of 10 μL in each well. After the cells were washed with PBS, the culture medium was replaced with fresh DMEM/F12 containing 10% CCK-8. The cells were further cultured at 37°C for 2 hours, and the OD value was measured at 450 nm with a micrometer.

- 1. Fluorescein Sodium Staining

1% fluorescein sodium solution was applied to the eye surface of healthy mice and left for 1 minute. The excess fluorescein sodium solution was sucked up with a cotton swab, and corneal fluorescein staining was observed under a slit lamp and recorded. Then, diverse treatment regimens, namely Ctrl (PBS), PDA (PDA NPs, 300 μg mL^-1^, NIR), EPL (EPL, 1%) and EPL@PDA (EPL@PDA NPs PBS solution, 300 μg mL^-1^, NIR) were conducted. After 24h, mice eyeballs were stained with fluorescein sodium and photographed again.

1. Results


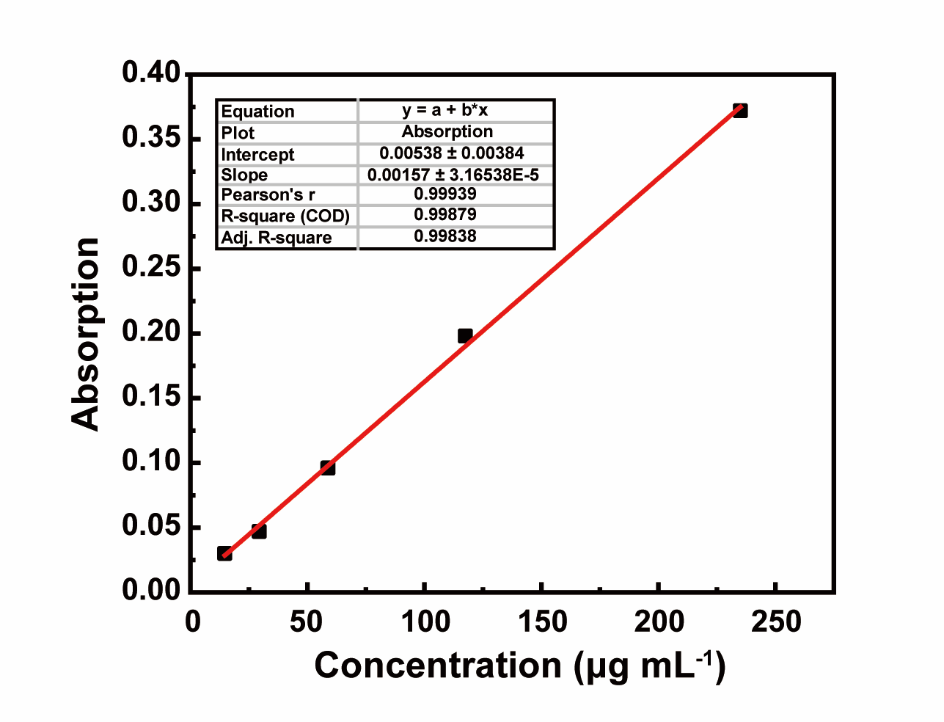


Figure S1 UV-visible standard operating curve of EPL@PDA NPs.


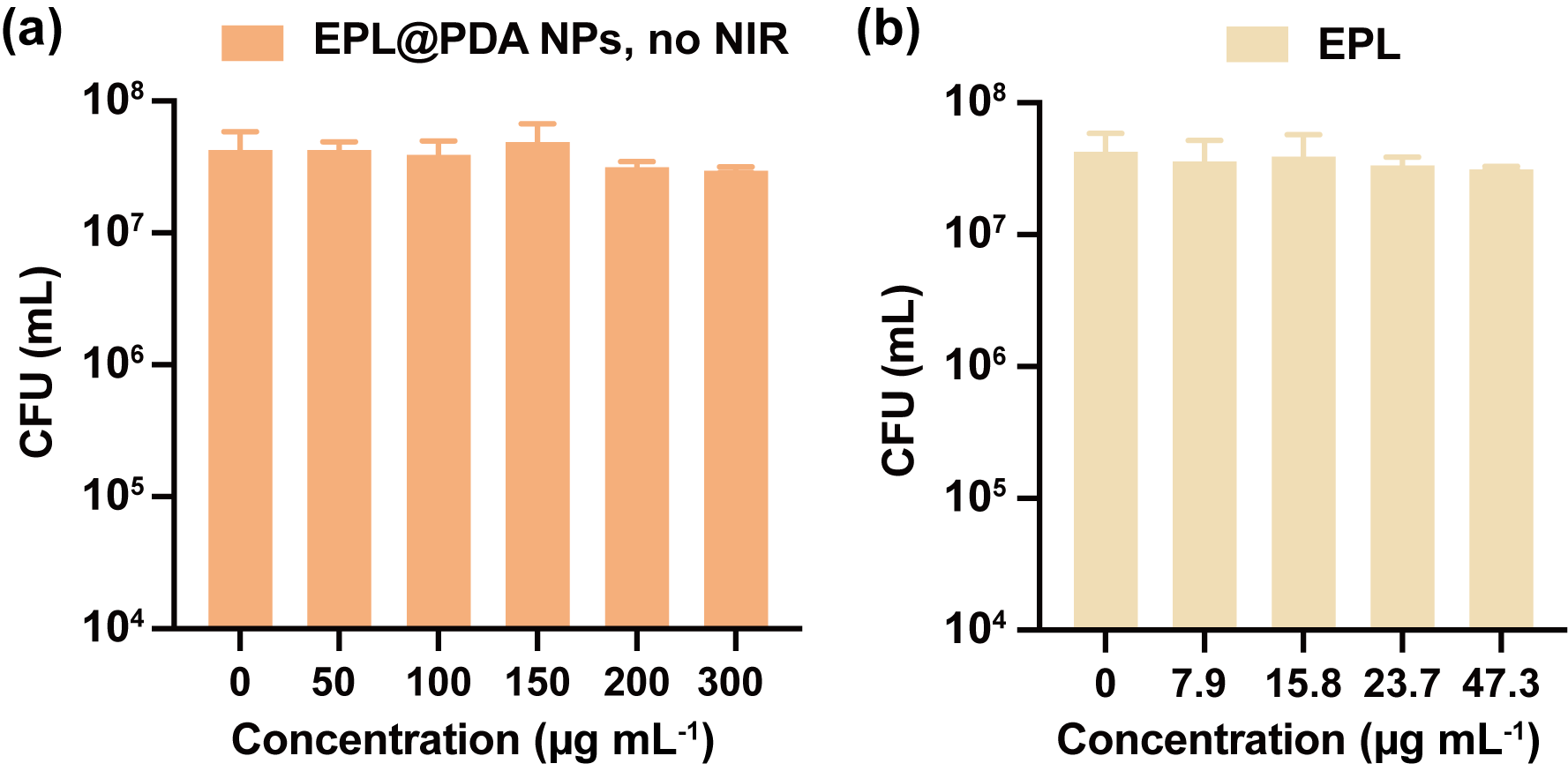


Figure S2 *In vitro* bactericidal performance of (a) EPL@PDA NPs and (b) EPL.


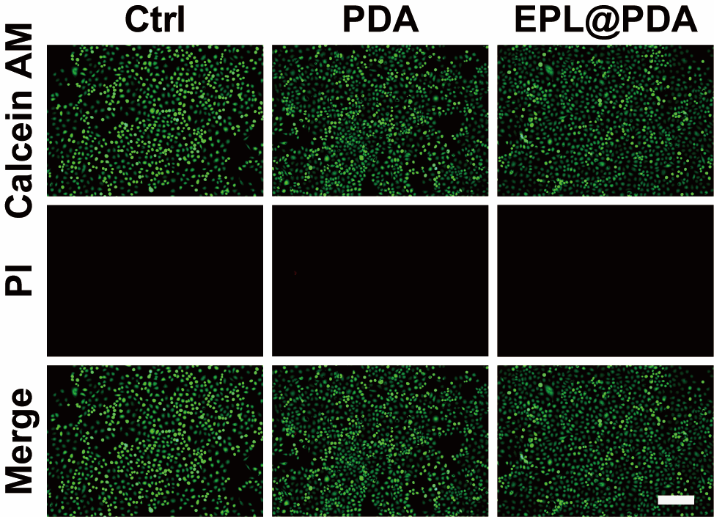


Figure S3 Cytotoxicity assessments of PDA and EPL@PDA NPs via calcein AM/PI staining. The concentrations of both PDA and EPL@PDA NPs were 400 μg mL^-1^. Scale bar: 200 μm.


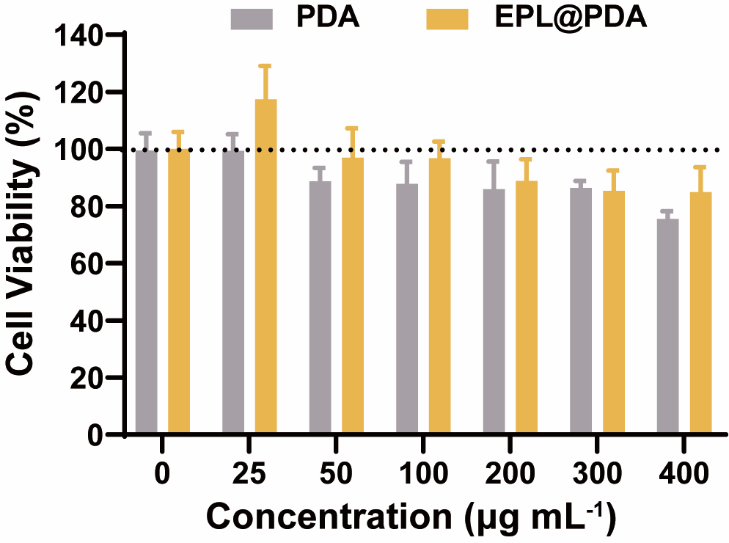


Figure S4 Cytotoxicity assessments of PDA and EPL@PDA NPs via CCK-8 analysis, in a series of concentrations ranging from 0 to 400 μg mL^-1^.


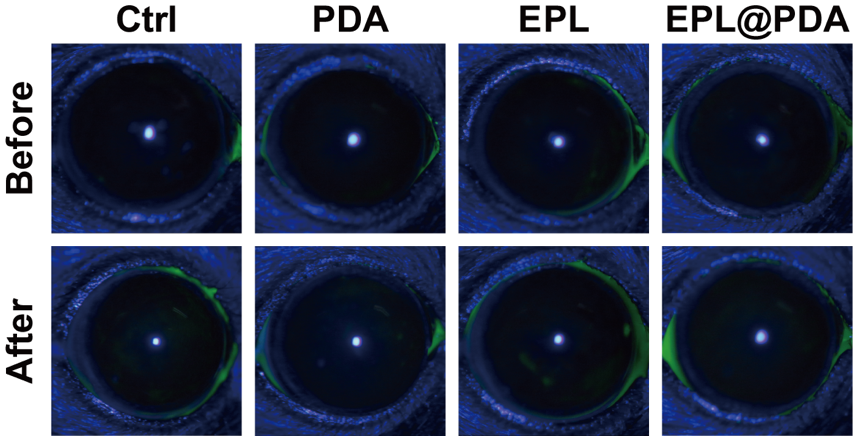


Figure S5 Fluorescein sodium staining on the healthy murine ocular surface before and after various treatments, namely Ctrl (PBS), PDA (PDA NPs, 300 μg mL^-1^, NIR), EPL (EPL, 1%), and EPL@PDA (EPL@PDA NPs solution, 300 μg mL^-1^, NIR). The wavelength of NIR light is 808 nm (2.55 W cm^-2^, 5 min).
